# Recent trends and biases in mesophotic ecosystem research

**DOI:** 10.1098/rsbl.2024.0465

**Published:** 2024-12-18

**Authors:** Veronica Z. Radice, Alejandra Hernández-Agreda, Gonzalo Pérez-Rosales, Ryan Booker, Jessica Bellworthy, Manon Broadribb, Gaby E. Carpenter, Clara Diaz, Ryan J. Eckert, Nicola L. Foster, Johanna C. Gijsbers, Erika Gress, Jack H. Laverick, Valerio Micaroni, Miriam Pierotti, Héloïse Rouzé, Angela Stevenson, Alexis B. Sturm, Pim Bongaerts

**Affiliations:** ^1^ California Academy of Sciences, San Francisco, CA, USA; ^2^ Department of Ocean Science, The Hong Kong University of Science and Technology, Clear Water Bay, Kowloon, Hong Kong; ^3^ Woods Hole Oceanographic Institution, Woods Hole, MA, USA; ^4^ Global Underwater Explorers, Queensland, Australia; ^5^ Department of Marine Biology, The Leon H. Charney School of Marine Sciences, University of Haifa, Haifa, Israel; ^6^ School of Biological Sciences, Victoria University of Wellington, Wellington, New Zealand; ^7^ Department of Oceanography and Coastal Sciences, Louisiana State University, Baton Rouge, LA, USA; ^8^ School of Biological and Marine Sciences, University of Plymouth, Plymouth, UK; ^9^ Harbor Branch Oceanographic Institute, Florida Atlantic University, Ft. Pierce, FL, USA; ^10^ Centro Interdisciplinario de Investigación para el Desarrollo Integral Regional (CIIDIR), Unidad Oaxaca, Instituto Politécnico Nacional, Santa Cruz Xoxocotlán, Oaxaca, México; ^11^ College of Science and Engineering, James Cook University, Townsville, Queensland, Australia; ^12^ Department of Mathematics and Statistics, University of Strathclyde, Glasgow, UK; ^13^ Department of Biological and Environmental Sciences and Technologies, University of Salento, Lecce, Italy; ^14^ Marine Laboratory, University of Guam, Guam; ^15^ Marine Biological Association of the UK, Plymouth, UK; ^16^ Marine Evolutionary Ecology, GEOMAR Helmholtz Centre for Ocean Research, Kiel, Germany; ^17^ National Ocean Service, National Centers for Coastal Ocean Science, National Oceanic and Atmospheric Administration (NOAA), Silver Spring, MD, USA

**Keywords:** mesophotic coral ecosystems, temperate mesophotic ecosystems, twilight zone, research biases, data repository, open access

## Abstract

Mesophotic ecosystems (approx. 30–150 m) represent a significant proportion of the world’s oceans yet have long remained understudied due to challenges in accessing these deeper depths. Owing to advances in underwater technologies and a growing scientific and management interest, there has been a major expansion in research of both (sub)tropical mesophotic coral ecosystems and temperate mesophotic ecosystems. Here, we characterize the recent global trends in mesophotic research through an updated release of the ‘mesophotic.org’ database (www.mesophotic.org) where we reviewed and catalogued 1500 scientific publications. In doing so, we shed light on four major research biases: a gross imbalance in (a) the geographical spread of research efforts, differences in (b) the focal depth range and (c) research fields associated with study organisms and research platforms, and (d) the lack of temporal studies. Overall, we are optimistic about the future of mesophotic research and hope that by highlighting current trends and imbalances, we can raise awareness and stimulate discussion on the future directions of this emerging field.

## 1. Main text

Over the past two decades, the interest in mesophotic ecosystems has transformed from an obscure research topic to an established body of research. This evolution was set in motion through an international workshop organized in 2008 by the U.S. National Oceanic and Atmospheric Administration (NOAA), where the term ‘mesophotic coral ecosystems’ (MCEs) was formally adopted, and the collection of associated research started to be tracked through the mesophotic.org database [1–3]. Now, celebrating the 15th anniversary since the establishment of the mesophotic.org repository, we provide an updated release of the database hosting a total of 1500 relevant scientific publications [4]. This update reflects a 4.2-fold increase in the body of literature on MCEs since 2008, and an even greater increase (6.8-fold) in research specific to temperate mesophotic ecosystems (TMEs). Despite ongoing discussions of terminology [5–7]—particularly given the breadth of biological communities found at mesophotic depths—the mesophotic definition has certainly helped focus and fund research on these understudied depths. The research topic has matured to the extent that there is a dedicated mesophotic session at most coral reef conferences, including the International Coral Reef Symposium [8], plus multiple Gordon Research Conferences and a dedicated volume of the ‘Coral Reefs of the World’ book series [9]. However, despite the major strides that have been made in our understanding of mesophotic ecosystems worldwide [2,5,10–14], an assessment of the database and major trends in geographical coverage, scientific discipline, taxa and research platform also reveal substantial biases. Given the ongoing climate crisis and increasing impacts of anthropogenic disturbances on marine ecosystems, we feel that there is urgency in addressing these biases as we move forward into the next decade of mesophotic research.

### (a) Broad geographical coverage but shallow understanding

**Figure 1.**
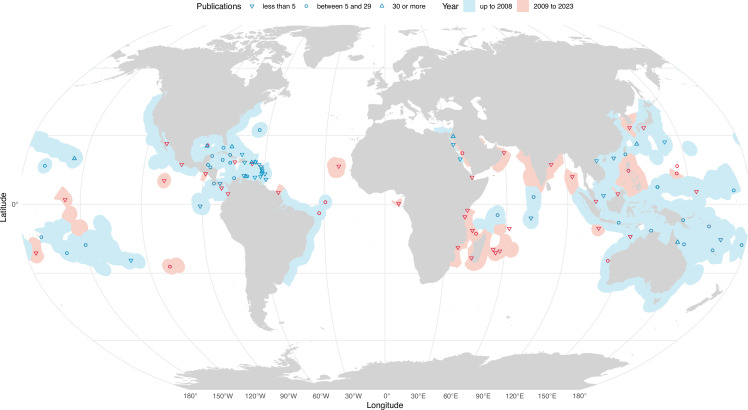
Worldwide distribution of mesophotic coral ecosystem (MCE) research and its expansion over the last 15 years. Exclusive economic zones [15] are coloured based on research locations from before 2008 (blue) and new locations based on publications from 2009 to 2023 (red). The shape indicates the number of publications per location (downward triangle—less than five publications, circle—between five and 29 publications, and upward triangle—30 or more publications). Only articles with original data are included (i.e. reviews were excluded). See the global distribution of temperate mesophotic ecosystem (TME) research in the electronic supplementary material, figure S1.

With the increased interest in mesophotic ecosystems, there has been nearly a doubling of unique research locations (i.e. geographical regions) since 2008, providing a much greater global coverage of these ecosystems. In the last 15 years, the number of unique locations increased from 66 to 111 MCE locations and from 19 to 42 TME locations (figure 1 and electronic supplementary material, figure S1). Nonetheless, there is an evident imbalance in the extent of research focus across locations. Nearly half of these locations only have one or two publications (43 MCE and 19 TME locations), while 65 out of 111 (59%) MCE locations and 26 out of 42 (62%) TME locations have five or fewer publications (electronic supplementary material, table S1). In contrast, the TME location Italy (Ligurian–Tyrrhenian Seas) has more than 90 publications and Spain (western Mediterranean Sea) has more than 30 publications. MCE locations USA–Hawai’i and Israel–Red Sea each have more than 90 publications and eight other MCE locations have more than 30 publications. The concentration of major research efforts in relatively few locations appears to stem from targeted funding that facilitated the development of mesophotic research infrastructure at established field sites. The two most extensively studied MCE locations are not necessarily representative of broad geographical areas but are rather unique: Hawai’i hosts a very high proportion of endemic species [16,17], and Israel–Red Sea hosts specialized assemblages that appear more resilient to warming due to the geological structure and climatic history of the Gulf of Aqaba [18,19]. Furthermore, most of the remaining highly studied MCE locations are located in the Caribbean Sea, which hosts distinct and much less biodiverse assemblages compared with the Indo-Pacific Oceans [20,21]. As capacity and interest grow, research is gradually extending to remote and less-resourced areas, with the small number of publications from these regions reflecting either their recent infrastructure establishment or, more commonly, the sporadic nature of exploratory expedition work. There are particularly few mesophotic research studies from the Indian Ocean (6.5% of all publications), as well as the Coral Triangle region, despite being biodiversity hotspots and having immense biological importance. Given this disproportion in research locations and the vast underrepresentation of more biodiverse areas, our current knowledge of mesophotic ecosystems is biased towards lower diversity regions. Overall, it is apparent that despite an increase in the geographical coverage of mesophotic research, our understanding of these ecosystems across most locations remains highly superficial.

### (b) The great divide: scleractinian corals and fish

**Figure 2.**
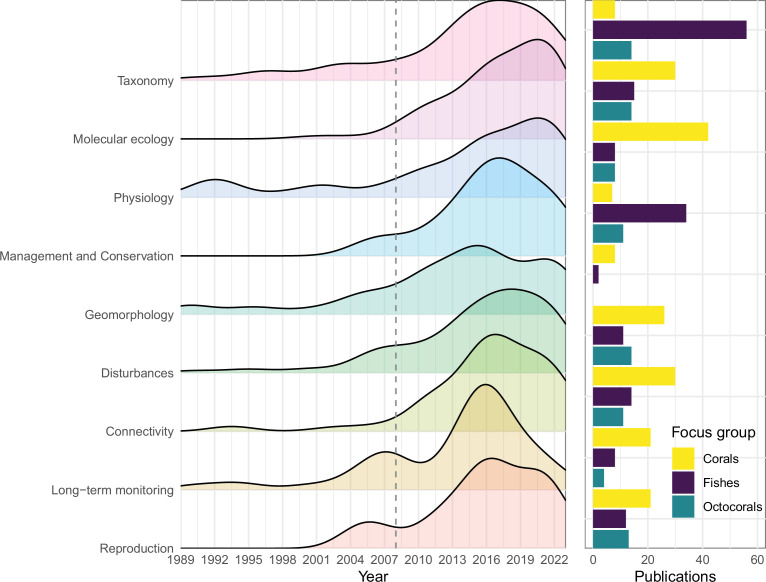
Temporal trends of emerging research fields in mesophotic science (MCEs and TMEs) and associated publication counts for the top studied organismal focus groups (scleractinian corals—yellow, fishes—purple and octocorals—green). Density plots display the emerging research fields over time (i.e. excluding the top three and more established research fields of biodiversity, ecology and community structure), with the order (top to bottom) representing the highest to lowest total publication counts for these fields (relative scaling calculated for each field of study). The dashed line marks 2008, to highlight changes in the last 15 years. Although density plots always show a drop at their edges, there was a peak in publication numbers in 2016 (the same year as the International Coral Reef Symposium or ‘ICRS’).

With increasing depth, the number of publications declines, and this pattern has remained relatively stable since 2008 [3]. However, when examining the two primary focal taxa, fishes and scleractinian corals, there is a stark difference in the depth coverage of these publications (electronic supplementary material, figure S2). Despite the substantial representation of fish literature across the entire mesophotic depth gradient, and well into mesophotic depths (over 60 m), the literature on scleractinian corals is overrepresented at 30−60 m with a major peak between 30 and 40 m depth and fewer studies beyond 60 m depth. While taxonomy and species discovery is a major research focus for mesophotic fishes (56 taxonomy articles with 54 describing new species), only eight articles focused on mesophotic scleractinian coral taxonomy (i.e. four new species described in total) and 15 articles focused on octocoral taxonomy (figure 2). Two emerging research fields in the study of mesophotic ecosystems, physiology and connectivity, have gained considerable traction for focus groups such as scleractinian corals (109 and 53 articles, respectively) and their algal endosymbionts Symbiodiniaceae (62 and 18 articles, respectively) but are less studied topics in fish (11 and 17 articles, respectively; figure 2). In summary, the coral literature is biased towards shallower mesophotic depths, with a greater emphasis on environmental gradients and connectivity over depth, while the fish literature focuses on biodiversity and species discovery in deeper parts of the mesophotic range. The limited number of publications inclusive of both focal taxa (16 publications) confirms the separation between coral and fish studies. Other taxonomic groups still remain vastly understudied in comparison (electronic supplementary material, figure S3) despite their abundance and perceived ecological importance across mesophotic depths (e.g. sponges, macroalgae, and Antipatharia). Possible reasons for the depth bias in corals versus fish may include the more limited depth distribution of light-dependent corals (decreasing in absolute and relative abundance with depth) and a more robust and established fish taxonomy versus the major challenges plaguing coral taxonomy, complicating the identification of new species. Further, logistical challenges related to deep diving limit the type of work typically done at greater depths (e.g. new species discoveries can be done through deep ‘bounce’ dives with short bottom times, whereas connectivity and physiology studies generally require more extensive bottom times). Broadly, the discrepancy in fish and coral studies has led to different perceptions, where the former being biased towards lower mesophotic depths and biological uniqueness, and the latter towards upper mesophotic depths and potential similarities (in comparison to shallow-water ecosystems).

### (c) Platform access matters

**Figure 3.**
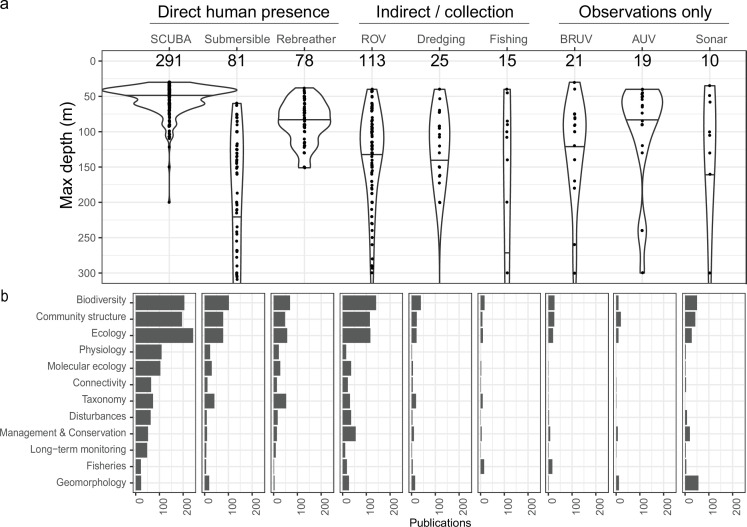
Differences in maximum study depths and research fields of by research platform. (*a*) The distribution of maximum study depths of all publications reporting the use of a particular research platform, and (*b*) the number of publications for each research field across research platforms. Research platforms are divided into ‘Direct human presence’, ‘Indirect human presence/Collection’, and ‘Observations only’. Acronyms stand for: SCUBA, self-contained underwater breathing apparatus; ROV, remotely operated vehicle; BRUV, baited remote underwater video; and AUV, autonomous underwater vehicle. Both (*a*) and (*b*) represent only publications with original data (e.g. reviews are excluded) and operating depths within the mesophotic range (i.e. minimum depth ≥ 30 m and maximum depth ≤ 150 m). Violin plots display the maximum depths of publications for a single platform but with a deeper maximum depth cut-off at 300 m for visual representation (i.e. fishing box plot goes beyond the *y*-axis because some publications within the mesophotic range had deeper maximum depths). Quantiles (0.5) inside violin plots display the median. Note: The platform SCUBA (open-circuit or unspecified) included 10 publications that indicated diving at depths greater than 100 m based on method details of the publication.

Early studies of mesophotic research often involved deep SCUBA diving using air [6]. Despite safety limitations in depth and bottom time, SCUBA remains a popular platform for upper mesophotic depths. There has been a steady adoption of newer technologies such as closed-circuit rebreathers, which had only seven reported uses in scientific articles until 2007, and are now reported in more than 170 scientific articles and have been used in 60 MCE and TME locations (about one-third of all locations). The use of remotely operated vehicles (ROV), and to a lesser extent, autonomous underwater vehicles (AUV), has also increased, while submersibles remain the top platform for studies in the lower mesophotic range (figure 3*a*). Despite the increased use of technology and diversity in platform types, explicit biases have emerged because scientists are frequently limited to particular platforms (e.g. due to funding or local scientific diving restrictions). First, platform type is an important determinant of the studied depth range due to the vastly different operating depths. The typical operational depths extend from regular SCUBA (approx. 30–40 m) to closed-circuit rebreathers and small inspection-class ROVs (approx. 60–100 m), to working-class ROVs and manned submersibles reaching well beyond 100 m depth (figure 3*a*). Secondly, operational platform limitations dictate the research fields possible, given that certain platforms are exclusively visual (e.g. allowing only for observational biodiversity and community structure studies) versus platforms that enable collections or other direct interventions (e.g. allowing physiological, genetic, or taxonomic studies; figure 3*b*). The platforms that support direct human presence (SCUBA diving, rebreathers) allows for the physical collection of diverse data, underwater experimentation, and *in situ* characterization of species biology. In contrast, collections remain more limited across platforms that only support indirect human access (ROV, dredging, and fishing). Consequently, fewer physiology and genetic studies have been undertaken using these platforms (figure 3*b*). Finally, the observational platforms, represented by AUV, baited remote underwater video (BRUV), and sonar, are typically limited to observational biodiversity and community structure studies. Although one would ideally choose the platform depending on the question or research field, the reality is that some of the most versatile platforms for studying mesophotic ecosystems (e.g. rebreathers, AUVs and submersibles) remain extremely costly, logistically complex and not accessible to the majority of researchers.

### (d) Snapshot fallacy: looking at one point in time

The vast majority of mesophotic studies still represent observations of a single point in time, with temporal monitoring being exceptionally rare. Not unexpectedly, long-term monitoring (i.e. over multiple timepoints) efforts are largely associated with geographical locations that are highly studied in the scientific literature (e.g. Israel–Red Sea, U.S. Virgin Islands, and Curaçao). Programmes such as the NOAA Coral Reef Conservation Program’s National Coral Reef Monitoring Program have produced 16 mesophotic long-term monitoring publications (articles and reports) from the U.S. Virgin Islands and Puerto Rico, and similarly for NOAA-funded mesophotic research in Pulley Ridge and the Gulf of Mexico (11 publications), showing the importance of consistent funding to support long-term monitoring research at mesophotic depths. Some of these aforementioned locations have established research stations, facilitating long-term monitoring of mesophotic ecosystems given the proximity to dive sites, staff support, and facility access (e.g. similarly for deep-sea research [22]). Although there are more articles and reports about temporal monitoring of the benthos, including scleractinian corals, Symbiodiniaceae, Octocorallia, macroalgae, and overall benthic groups (97 total) than fishes (23 total), there are more articles discussing fisheries and also management and conservation of fishes than other groups, likely due to widespread regulations and monitoring related to commercial fisheries (figure 2). The almost complete lack of decadal monitoring studies at mesophotic depths means that rather than a ‘shifting baseline syndrome’, the field is completely biased towards the observations recently made, well into the Anthropocene. Already, a handful of studies have highlighted the major changes that deeper reef communities have undergone in the recent past [23–27]. While obtaining an accurate picture of what mesophotic reefs would have looked like several decades ago has become almost impossible, they will undoubtedly continue to face major changes hence prioritizing long-term monitoring efforts is paramount. Understanding these changes is critical in order to develop and implement conservation measures that extend beyond shallow waters, with consideration of the unique properties of biological communities at mesophotic depths [28–30].

## 2. Conclusions

Over the past years, we have seen major strides in advancing our understanding of mesophotic ecosystems and generating public awareness about them—achievements truly worth celebrating. However, while reflecting upon the current trends based on published literature, there are also major biases that come to light, four of which we have highlighted in this perspective. The geographical bias highlights that despite our broadening geographical spread, most of our knowledge comes from a few focal locations that are not necessarily representative of larger geographical areas and mostly from tropical MCEs. Besides ensuring that we increase efforts in poorly studied locations, we should strategically broaden our geographical scope to include locations that more broadly represent mesophotic ecosystems across large biogeographical regions as well as key biodiversity hotspots, such as the Coral Triangle. The perceptual bias of how fish and coral researchers view ‘mesophotic ecosystems’ warns us against binary characterizations, encouraging us to be more specific and nuanced when drawing conclusions regarding the wide environmental gradient across which mesophotic communities occur. Given the substantial cost associated with many of the technologies used to access mesophotic environments, there should be more efforts to make these more widely available, particularly to lower income countries, and to organize multidisciplinary research expeditions. Similarly, sustained funding and local capacity building to enable temporal studies should be prioritized to better understand and monitor how mesophotic reefs are changing in response to increasing stressors and how these threats may be potentially mitigated. These are only some of the biases that exist in this newly emerged research field, and we hope that the release of the updated mesophotic.org database will enable further reflection on our current state of knowledge.

## Data Availability

The mesophotic.org online data repository is open access and available on the 'Publications' page with advanced search capabilities and the ability to download a csv file of the current version (www.mesophotic.org/publications). The source code (website) and code for the analyses and figures (R markdown files) are open access (https://github.com/pimbongaerts/mesophotic/). A static copy of the database used in the analyses (publications through 2023, inclusive) can be found on the GitHub page and also available from the Dryad Digital Repository [4]. Supplementary material is available online [31].
